# Fewer losses in the cascade of care for latent tuberculosis with solo interferon-gamma release assay screening compared to sequential screening

**DOI:** 10.1186/s12879-021-06637-z

**Published:** 2021-09-09

**Authors:** R. K. Lim, R. Talavlikar, O. Chiazor, J. Bietz, H. Gardiner, D. Fisher

**Affiliations:** 1 Cumming School of Medicine, University of Calgary, Alberta, Canada; 2 Mosaic Refugee Health Clinic, Mosaic Primary Care Network, Calgary, AB Canada; 3 Calgary Tuberculosis Services, Alberta Health Services, Calgary, AB Canada

**Keywords:** Tuberculosis, Latent tuberculosis, Screening, Refugee

## Abstract

**Background:**

Refugees are at increased risk of developing tuberculosis (TB) soon after resettlement. Targeting high-risk populations for latent tuberculosis infection (LTBI) screening and treatment is an important measure towards eliminating TB in low incidence countries, however, there are low rates of screening and treatment completion in the LTBI cascade of care. The authors hypothesized that an interferon-gamma release assay (IGRA) screening strategy would lead to a higher proportion of refugees completing LTBI screening and treatment, compared to sequential screening with tuberculin skin test (TST) and confirmatory IGRA.

**Methods:**

This retrospective cohort study included eligible refugees screened with a sequential strategy versus a solo-IGRA strategy at different time periods from a centralized refugee clinic. The primary outcome was the proportion completing LTBI screening in each cohort.

**Results:**

A total of 471 subjects were included (240 in sequential screening, 231 in solo-IGRA screening). 54% of refugees completed LTBI screening with sequential testing, compared to 85% of those screened with a solo-IGRA. Time to completing screening was also shorter in the solo-QFT group (difference 16.5 days, p < 0.01, 95% confidence interval 9.3, 23.7). There was a higher incidence of LTBI diagnosis in the solo-IGRA group (41 versus 20, p = 0.002). Screening completion was predicted by solo-IGRA screening (aOR 3.74, 95% confidence interval 2.30, 6.09; p < 0.001) and if refugees were privately-sponsored (aOR 2.81, 95% confidence interval 1.53, 5.15; p = 0.001). Treatment completion rates did not differ between groups.

**Conclusion:**

This study has identified fewer dropouts in the LTBI cascade of care if a solo-IGRA strategy is used for screening. An IGRA should be strongly considered as the screening method for refugees arriving in low-incidence settings if resources are available.

## Introduction

It is estimated that 25% of the global population is infected with latent TB, of which approximately 5–10% are expected to develop reactivation TB in their lifetimes [1]. With increasing global migration, international migrants continue to carry the majority of the TB disease burden in many low-incidence countries [2, 3]. In Canada, foreign-born individuals accounted for 71.8% of TB cases in 2017 [4]. Screening for active TB is currently mandatory for certain populations upon entry into Canada, while LTBI screening is recommended [2, 5]. The current Canadian guidelines on LTBI screening recommend either test, however, many centers that use the tuberculin skin test (TST) as an initial test will pursue confirmation with the more specific interferon-gamma release assay (IGRA) [6]. This sequential screening strategy has been shown to reduce the number of individuals recommended for LTBI treatment presumably due to false-positive results from a TST [7–9]. More recently, a combined societal guideline in the United States has recommended an IGRA over a TST in those with suspected infection at low to moderate risk of disease progression, although the TST remains an acceptable alternative [10]. The IGRA as a single screening test is relatively novel in the refugee population. According to a systematic review on the prevalence of LTBI among refugees and asylum seekers, the majority of cohort studies (18) published after 2010 utilized both TST and IGRA for diagnosis of LTBI, compared to seven studies that used only TST and three studies that used an IGRA alone [11].

Low completion rates of LTBI screening and treatment (herein referred to collectively as the LTBI cascade of care) threatens the effectiveness of TB preventive efforts. A systematic review determined that the losses in the LTBI cascade of care are substantial and most of the drop-outs occurred before treatment was even initiated [12]. Among migrants with LTBI, the proportion achieving treatment completion was only 14% [12]. The LTBI cascade of care involves multiple steps including eligibility determination, completing test(s), offering treatment, initiation, and completing LTBI therapy. Unfortunately, this entire process can be difficult to complete, and losses can occur at every step. The evolution of LTBI treatment to include shorter, more tolerable regimens has helped to improve treatment compliance [13, 14]. However, strategies to improve compliance during earlier steps in the cascade of care are desperately needed.

The TST and IGRA are the currently available diagnostic tests for LTBI. TST screening has been used for decades but cross-reactivity with the Bacille Camille-Guerin (BCG) vaccine can lead to the overdiagnosis of LTBI, particularly among migrants from countries where BCG vaccination is universal. Advantages of IGRAs include less cross-reactivity with the BCG vaccine and non-tuberculous mycobacterium, as well as being a one-time blood test, however, it is more costly compared to a TST [15]. Despite current guidelines, many programs may still be using sequential screening due to the lower costs of a TST, but the disadvantages of this strategy include the number of steps to completion. We hypothesized that an IGRA-based strategy would lead to higher completion rates of LTBI screening and treatment.

In Calgary, Alberta, newly arrived refugees are assessed at the Mosaic Refugee Health Clinic (MRHC) for comprehensive primary care. Sequential screening with an initial TST and confirmatory IGRA was replaced by IGRA screening in 2019. In this retrospective cohort study, we describe and compare the LTBI cascades of care with each screening modality.

## Methods

We conducted a retrospective cohort study of refugees seen at the Mosaic Refugee Health Clinic in Calgary, Alberta. Refugees arriving in Canada come as privately-sponsored refugees (funded by family and private organizations), government-assisted refugees (funded by the federal government), or refugee claimants (who make an asylum claim at or after arrival in the country). Medical care including testing and treatment related to this study were completely funded by the Interim Federal Health Program. The MRHC is a multidisciplinary specialized primary care clinic responsible for assessing all refugees in Calgary, typically within one month of arrival. Initial assessment of refugees includes LTBI screening if they are aged ≤ 50 years, or if they are older with comorbid conditions, as per national guidelines. During the study period, the sequential method of screening was done by an initial TST, followed by a confirmatory IGRA if the TST was positive (≥ 10 mm induration). The IGRA used was the QuantiFERON-TB Gold Plus (QFT) (Qiagen). QFT results were classified as positive, negative, or indeterminate according to the manufacturer’s specifications. The QFT was drawn at a local laboratory located about four kilometers away from the MRHC during the sequential screening period and the first three months of the QFT screening period, before it was routinely done at the MRHC along with other routine lab work.

The time period assessing the sequential LTBI screening method included individuals assessed at MRHC between August 2017 and December 2018. This method was replaced by QFT screening in March 2019 and individuals were included in the comparison cohort between March 2019 and February 2020. In both cohorts, a positive QFT triggered a chest radiograph and referral for LTBI treatment at the local TB clinic. Individuals were screened for active TB before being recommended LTBI treatment. Individuals deemed eligible for LTBI treatment at the TB clinic were offered one of the following regimens according to local practice and patient factors: three months of isoniazid and rifampin (3HR), four months of rifampin (4R), or nine months of isoniazid (9H). Treatment initiation was defined as at least one dose being taken. Treatment was considered complete after at least 80% of doses were taken within 120% of the recommended duration. Subjects were excluded if they were under the age of 2 years given that this age group is only screened with a TST in our setting, if they had active TB at the time of their initial assessment, and if they had previously been treated for TB or LTBI before immigration.

Demographic factors were recorded for all subjects. The completion rates were determined at each step of the cascade of care for both cohorts, including the initial intake appointment when eligibility for LTBI screening is determined, steps to completing LTBI screening, follow-up medical evaluation of those diagnosed with LTBI, LTBI treatment initiation, and treatment completion. Data was obtained from MRHC which tracked subjects as they completed each step of LTBI screening. Data regarding LTBI treatment eligibility, initiation, and completion were collected from the electronic database at the local TB clinic, which provides all TB-related care and medications for southern Alberta. Primary outcomes of interest were the proportion of individuals completing LTBI screening and time to screening completion. Other outcomes of interest were the proportion of those diagnosed with LTBI who initiated and completed treatment and factors associated with screening completion and treatment initiation. Patient consent was waived due to the retrospective nature of the study and ethics approval was obtained from the University of Calgary Conjoint Health Research Ethics Board. The study was conducted in accordance with the STROBE guidelines on observational studies. [16].

The cohort characteristics were summarized with means for continuous variables and frequencies for categorical variables, respectively. We calculated proportions completing each step of the LTBI screening and treatment cascade. We used a Chi-square test to compare categorical variables and a two-sample *t*-test to compare the means of continuous variables. We also used univariate and multivariate logistic regression to identify predictors in screening and treatment completion. Statistical analysis was done using STATA 16.1 software (StataCorp LP, College Station, USA).

## Results

### Population demographics

**Fig. 1:**
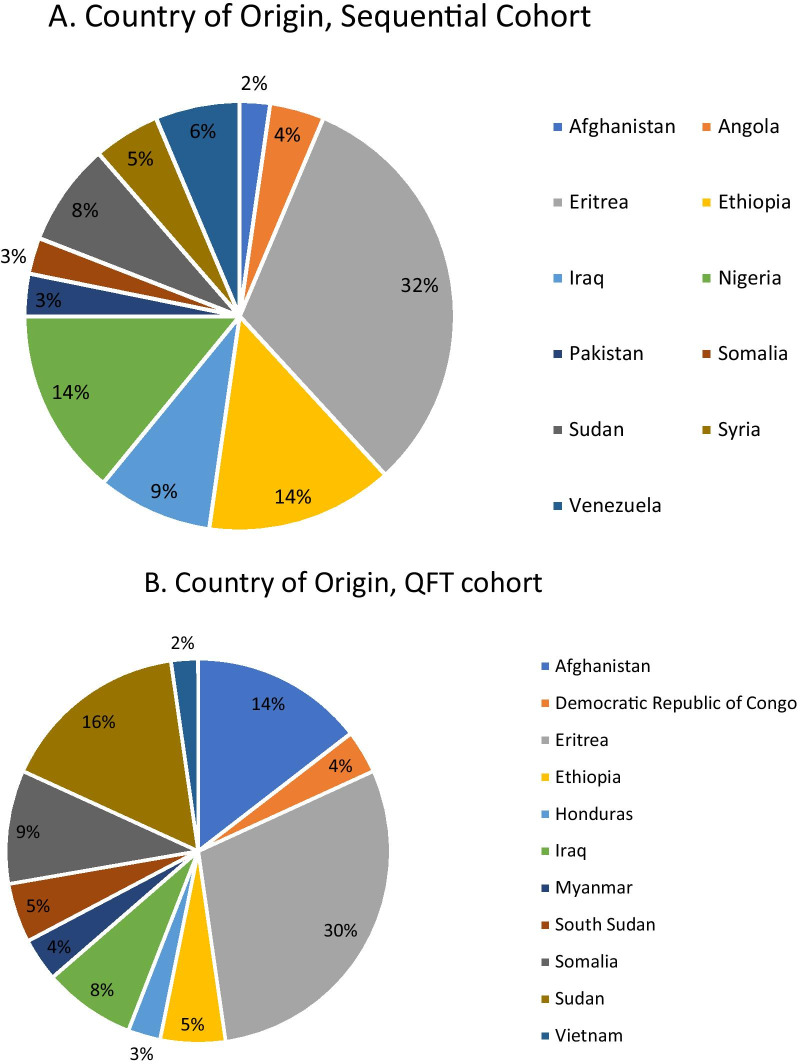
Countries of origin by screening strategy

There were 471 individuals in this cohort study, with 240 subjects in the sequential screening cohort and 231 subjects in the solo-QFT screening cohort. Thirteen individuals were excluded because they were either less than 2 years old or had active TB on the initial assessment. Demographics are outlined in Table 1. The mean age in the overall population was 24.8 years and 54% were males, with no significant differences between the two screening cohorts. The countries of origin of subjects in the cohorts are depicted in Fig. 1. The most commonly represented countries in the sequential screening cohort were Eritrea, Ethiopia, and Nigeria, accounting for 52% of refugees. In the solo-QFT cohort, Eritrea, Sudan, and Afghanistan were the most frequent countries of origin. There was an equal distribution of individuals originally from high TB incidence countries between cohorts (defined as greater than 100 cases per 100,000 population). There was a different composition of refugee statuses between groups, with fewer claimants and more privately-sponsored refugees in the solo-QFT group.

**Table 1:** Population demographics

	Sequential Screening N = 240	Solo Quantiferon N = 231	Entire Population = 471
Age, mean years	25.3	24.3	24.8
Gender			
% males	53%	55%	54%
Refugee status			
Claimant	88 (37%)	7 (3%)	95 (20%)
Private sponsored	83 (34%)	142 (62%)	225 (48%)
Government assisted	69 (29%)	82 (35%)	151 (32%)

### Screening completion rates

Within the sequential screening cohort, 54% (129 out of 240) completed LTBI screening. In comparison, 85% (197 out of 231) of the solo-QFT cohort completed screening. The difference in the proportion completing screening was 0.31, in favor of the solo-QFT strategy (p < 0.01). The mean number of days from the initial appointment to completing screening tests was 23.3 days (standard deviation 35.5) in the sequential screening group versus 6.8 days (standard deviation 26.8) in the solo-QFT screening group (difference 16.5 days, p < 0.01). QFTs were performed at the local laboratory for subjects in the sequential screening group as well as a minority of the solo-QFT group (50 of 231). Within the solo-QFT group, a lower proportion completed screening if they were instructed to go to a nearby local laboratory compared to those who had it drawn at the MRHC (p < 0.01).

In the univariate analysis, age and gender did not impact screening completion. Origin from a high-incidence country also did not impact screening completion (odds ratio 0.69; 95% CI 0.47, 1.03, p = 0.07). Privately sponsored refugees were more likely to complete screening compared to refugee claimants (odds ratio 5.7; 95% CI 3.36, 9.65, p < 0.01) and government-assisted refugees (odds ratio 2.37; 95% CI 1.47, 3.82, p < 0.01). In the adjusted analysis, solo-QFT screening and private-sponsored refugees were the only predictors of screening completion (see Table 2).

**Table 2:** Univariate and multivariate regression analyses of screening completion

	No. total	No. completing screening	OR (95% CI)	p-value^a^	aOR (95% CI)	p-value
Gender						
Male	255	176	Reference		Reference	
Female	216	150	1.03 (0.70,1.53)	0.871	1.10 (0.72, 1.69)	0.661
Refugee status						
Refugee claimant	95	42	Reference		Reference	
Privately-sponsored	225	185	5.69 (3.36, 9.65)	< 0.001	2.81 (1.53, 5.15)	0.001
Government-assisted	151	99	2.40 (1.42, 4.07)	0.001	1.38 (0.76, 2.49)	
Origin, by TB incidence						
Low/Intermediate incidence country	278	201	Reference		Reference	
High incidence country	193	125	0.69 (0.47, 1.03)	0.069	0.86 (0.55, 1.36)	0.524
Screening test						
Sequential	240	129	Reference		Reference	
Solo-QFT	231	197	4.84 (3.12, 7.52)	< 0.001	3.74 (2.30, 6.09)	< 0.001

No. number, OR odds ratio, CI confidence interval, aOR adjusted odds ratio, QFT quantiferon Gold Plus;

^a^Significant value defined as *p* ≤ 0.05; Log likelihood = − 250.43577, *p*-value final model = 0.000, No. of observations in final model = 467

In the sequential screening cohort, 20 subjects were diagnosed with LTBI, compared to 41 in the solo-QFT cohort (p = 0.002). Two subjects had an indeterminate QFT result, one in each cohort.

### Treatment outcomes for individuals with LTBI

Of the 20 individuals diagnosed with LTBI in the sequential screening cohort, 19 attended their appointment at the TB clinic. One individual was diagnosed with active TB two years after they were lost to follow-up. Another individual who presented for LTBI assessment was also diagnosed with active TB and two others were lost to follow-up. In total, 16 individuals began treatment, and 14 completed treatment.

Of the 41 refugees identified to have LTBI using the solo-QFT approach, 6 subjects did not attend their initial TB clinic appointment. Of 35 subjects, 33 started treatment, and 29 completed treatment. One individual moved provinces and the treatment outcome is unknown. Three subjects were lost to follow-up after treatment was initiated and another subject was lost to follow-up before treatment was initiated. There was no significant difference in treatment completion between cohorts (p = 0.8929).

The choice of treatment regimen for LTBI generally differed between cohorts, likely because of the publication of a landmark trial demonstrating non-inferiority of the 4R regimen compared to 9H in 2018 [9]. In the sequential screening cohort, eight subjects were treated with 3HR, three subjects with 9H, and five subjects with 4R. In the solo-QFT group, thirty subjects were treated with 4R, two with 9H, and one individual with HIV was treated with a one-month regimen of rifapentine and isoniazid. Sample sizes were too small to compare treatment completion rates among the different regimens.

### Cascades of care in LTBI screening and treatment

**Fig. 2:**
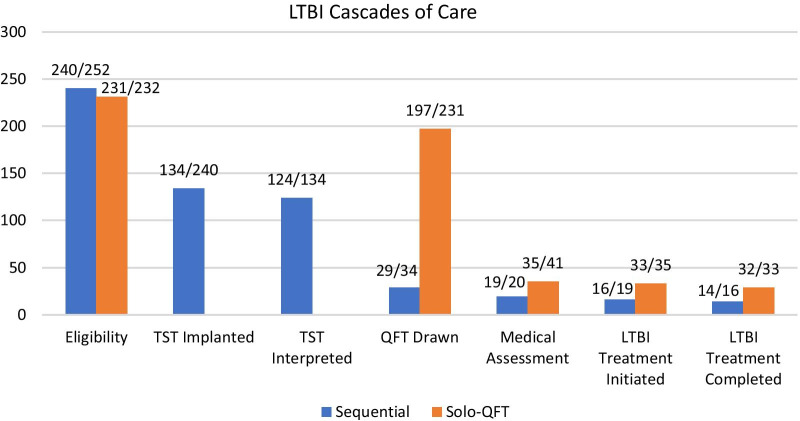
LTBI cascades of care among resettled refugees arriving in Calgary, Alberta. The total numbers of individuals who completed each step as a proportion of those eligible are labeled above the bars. For example, the population eligible for QFT was comprised of those with positive TST results in the sequential group. Similarly, only those with positive QFT results were eligible for medical assessment for LTBI treatment

Figure 2 showcases the LTBI cascades for the two cohorts. The major losses occurred between initial assessment and testing for LTBI. This loss was greater in the sequential screening group, as almost half did not have the initial TST implantation. There were also losses before having the TST read and having a confirmatory QFT. For those with LTBI, both cohorts had high attendance at the TB clinic, as well as good treatment outcomes.

## Discussion

To our knowledge, this is the first study to compare the real-world experience of a sequential screening method to a solo-QFT method among refugees. We identified more drop-outs in screening completion among refugees assessed by sequential TST and QFT testing compared to those assessed with a solo-QFT. The use of solo-QFT screening was the strongest predictor of completing screening in our analysis, with an adjusted OR of 3.74 (95% CI 2.30, 6.09; p < 0.001). This difference between cohorts represents a missed opportunity to reduce the risk of reactivation TB for a significant proportion of newly arrived refugees when sequential screening is used. As identified in a recent meta-analysis, many candidates for LTBI therapy are lost before they can be offered treatment, either by not undergoing screening or failing to complete screening [12]. Unfortunately, several host factors make refugees particularly vulnerable to TB infection, including malnutrition, crowded living conditions, and prior residence in higher incidence countries [17]. Migrants are also at the greatest risk of developing TB disease within the first 2 to 5 years of arrival, hence, there is an advantage to completing screening more quickly as was seen with the solo-QFT cohort [18, 19]. Improving the uptake of LTBI screening among high-risk populations is particularly salient, as the World Health Organization’s End TB strategy to eliminate TB by 2035 indicates a reduction of the large reservoir of those with latent TB as a core intervention [20].

Comparison between our two cohorts found that the largest differences were in screening uptake and completion. A major difference was seen in the lack of uptake in TST implants in the sequential screening group. TSTs were implanted on the same day as the refugee’s initial appointment and done at the clinic, therefore, convenience and travel are unlikely to have been major factors. One possibility is that the knowledge of having to return for the interpretation and having a blood test (IGRA) if the TST was positive may have seemed too prohibitive or burdensome for many new refugees. Although there were some losses to follow-up for subsequent steps in the cascade, the MRHC staff routinely ensure that appointment reminders and instructions are given in the refugee’s preferred language, which likely helped adherence in both cohorts. There were no differences in treatment initiation or completion among those diagnosed with LTBI, despite a variation in the treatment regimens. Similarly, there were no explicit differences in the provider counseling around LTBI screening or treatment. A key intervention to improve the LTBI cascade of care for the refugee population is to provide ease and access to LTBI testing (specifically referred to in some implementation interventions as a "one-stop shop”). In support of this is the observation that more subjects completed solo-QFT screening when it was done on-site at the refugee clinic compared to a nearby laboratory. In the sequential screening, refugees were required to go to the nearby laboratory for QFT instead of remaining at the clinic. Fortunately, 85% of those eligible still completed the test. Convenience and minimization of travel have been cited as key factors in completing LTBI therapy [21], and the same factors are likely to play a role in completing screening.

Another predictor of screening completion was being a privately-sponsored refugee. Previous studies have suggested that this group may have more financial and social supports compared to claimants, allowing them to better navigate the health care system [22]. Although this study was focused on the impact of simplifying the LTBI screening cascade with the use of an IGRA, programs that care for the refugee population should continue to explore other strategies to remove barriers to TB screening and treatment. After arrival into a new country, refugees are pursuing many resettlement tasks, such as finding employment, housing, and child care, making it difficult to prioritize health promotion activities like LTBI screening.

Confirmation of positive TST results with an IGRA consistently reduces the number of individuals referred for treatment [7–9], but we found a major limitation was the ability of individuals to complete the numerous steps of screening. As a result, more cases of LTBI were diagnosed in the IGRA group. A higher drop-out rate has been observed in other TB programs screening with a TST compared to an IGRA [23], yet TST-based approaches (either TST alone or sequential with confirmatory IGRA) are still more prevalent than IGRA screening in the literature [11]. One reason for this may be the cheaper cost of a TST compared to a QFT. However, cost-effectiveness studies of high-risk populations suggest that QFT-based screening may be more cost-effective, particularly when the prevalence of LTBI is high [24, 25]. Successful implementation of a solo-QFT strategy will ultimately depend on the availability of adequate equipment and trained lab personnel to draw fresh blood samples and perform the assays, as well as the infrastructure to avoid transportation delays of samples.

This study has limitations. Individuals were not randomized to a screening strategy and other unknown factors may have impacted the primary outcome. Potential confounders that could not be avoided include the different make-up of refugee classes between the cohorts, as well as the administration of the QFT test at the MRHC for most of the solo-QFT cohort. It is possible that the predominance of privately sponsored refugees in the solo-QFT cohort could be affecting the results, however, our multivariable analysis still found the use of solo-QFT screening to be an independent predictor of screening completion. Counseling practices on the importance of LTBI screening were not explicitly changed during either period, however, we cannot exclude a shift in health care worker attitudes or behaviors during the QFT screening period as they were not blinded to the screening test. Additionally, the study had a short follow-up time and we are unable to confidently report on the incidence of active TB cases following screening. Despite these limitations, our study found that utilizing a QFT screening strategy in refugees led to higher screening completion rates and as a result, more cases of diagnosed LTBI.

## Conclusion

TB elimination in low-incidence countries will require targeted interventions, including better assessment and treatment of refugees at risk for developing TB disease after resettlement. Our study has suggested that losses in the LTBI cascade are substantially decreased when using an IGRA-alone screening strategy compared to sequential screening, potentially due to a simplified process with fewer steps. An IGRA should be strongly considered for screening eligible refugees arriving in low-incidence countries if the resources are available. Further cost-effectiveness studies into this screening approach is warranted.

## Data Availability

Available upon request by email to corresponding author.

## Abbreviations

QFT: Quantiferon
